# A nationwide registry study of surgical and patient-reported outcomes following anterior cervical discectomy and fusion: Part 2 – cage with versus without local bone graft

**DOI:** 10.1007/s00701-025-06751-w

**Published:** 2025-12-11

**Authors:** Daniel de Wilde, Victor Gabriel El-Hajj, Patrick Vigren, Victor E. Staartjes, Elias Atallah, Erik Edström, Adrian Elmi-Terander

**Affiliations:** 1 Machine Intelligence in Clinical Neuroscience & Microsurgical Neuroanatomy (MICN) Laboratory, Department of Neurosurgery, Clinical Neuroscience Center, University Hospital Zurich, University of Zurich, Zurich, Switzerland; 2 Department of Clinical Neuroscience, Karolinska Institutet, 171 77 Stockholm, Sweden; 3 Department of Neurosurgery, Region Örebro County, Örebro University Hospital, Örebro, Sweden; 4 Capio Spine Center Stockholm, Löwenströmska Hospital, Upplands-Väsby, Sweden; 5 Department of Neurosurgery, Thomas Jefferson University Hospital, Philadelphia, PA USA

**Keywords:** Anterior discectomy and fusion, Cervical spondylosis, Patient reported outcome measures, Bone transplantation, Registry

## Abstract

**Background:**

Local bone grafts are commonly used as filling material in cages during anterior cervical discectomy and fusion (ACDF). Alternatively, cages without filling materials can be used. The literature comparing these approaches is limited, and their respective effects on patient-reported outcomes (PROM) have been scarcely studied. This study was conducted to compare surgical outcomes and PROMs between local bone graft-packed cages and empty cages in ACDF surgery.

**Methods:**

This observational study utilized data from the Swedish nationwide registry, Swespine. All adults who underwent ACDF between 2006 and 2020 were considered for inclusion. Exclusion criteria included missing baseline or outcome data. Patients were grouped according to cage type (with vs. without local bone graft). The primary outcome was achievement of a minimal clinically important difference (MCID) for arm pain in radiculopathy patients and for the European Myelopathy Score (EMS) in myelopathy patients. Secondary outcomes included complications, reoperations, and length of hospital stay. Outcomes were analyzed using multivariable generalized linear models adjusted for clinically relevant covariates.

**Results:**

A total of 6,571 patients were included, with 2,963 patients (45%) receiving cages with local bone graft and 3,608 patients (55%) receiving cages without local bone graft during ACDF. Achievement of MCID for arm pain (radiculopathy) (OR 0.90, 95% CI 0.79–1.04, *p* = 0.15) and for EMS (myelopathy) (OR 1.15, 95% CI 0.67–2.03, *p* = 0.62) did not differ between groups. Local bone graft use was associated with higher odds of reoperation (OR 1.69, 95% CI 1.20–2.39, *p* = 0.003) and a longer hospital stay (β = 0.31 days, 95% CI 0.21–0.40, *p* < 0.001).

**Conclusion:**

This nationwide registry-based study demonstrates that ACDF performed with or without local bone graft is equally safe and effective, with comparable rates of PROM MCID achievement and similar adverse event profiles, although use of local bone graft was associated with higher reoperations and longer hospital stays.

**Supplementary Information:**

The online version contains supplementary material available at 10.1007/s00701-025-06751-w.

## Introduction

Anterior cervical discectomy and fusion (ACDF) is a widely established spinal surgical procedure that has been in practice for over 60 years. With approximately 137,000 ACDF procedures performed annually in the United States alone, it is one of the most common spinal surgeries [26]. ACDF has proven effective for the long-term treatment of conditions such as disc herniation, cervical stenosis, and advanced degenerative disease, with patient-reported success rates ranging from 85 to 95% [4, 13]. The procedure involves anterior decompression through removal of osteophytes or intervertebral discs, followed by the insertion of a cage or allograft into the disc space to provide spinal support and facilitate segmental union [29].

To enhance fusion rates, additional filling materials, including autologous iliac bone grafts, demineralized bone matrix (DBM), or allografts, are often used [16, 29, 34]. Historically, iliac crest autografts have been the preferred choice, but this approach is associated with complications such as donor-site pain, hematomas, and infections [24, 27]. In contrast, non-autologous materials, while effective, often incur additional costs. To mitigate both the complications and the additional costs, cages filled with local bone grafts have emerged as a viable alternative [17, 18, 20, 21]. This method has demonstrated relatively high fusion rates and has gained widespread adoption [31, 35].

An alternative approach involves the use of cages that are implanted without any graft material [22, 23]. While both these techniques are commonly used, there is limited evidence comparing the outcomes of cages filled with locally harvested autologous bone versus empty cages, particularly in large, real-world patient populations. Most previous studies have primarily focused on radiological fusion rates or clinical outcomes in small cohorts, with little literature on patient-reported outcome measures (PROM) [17, 30].

This study aims to address this gap by evaluating both surgical and patient-reported outcomes in patients undergoing ACDF with local bone-packed cages versus those receiving cages without local bone graft material.

## Methods

### Study design and data source

An observational study was conducted to compare the effects of cages filled with versus without local bone grafts during ACDF in terms of surgical outcomes and PROMs. Patients who underwent ACDF between 2006 and 2020 were divided into two groups: those receiving local bone-packed cages and those receiving cages without local bone graft. Data for this study were obtained from the nationwide Swedish Spine Registry, Swespine [5, 7, 8, 28]. The Swespine registry covers 95% of the Swedish spine centers (*n* = 47), with 85% (50–95%) of the surgeries performed being registered [1]. This study is in accordance with STROBE guidelines.

### Variables

Demographic variables collected included age, sex, body mass index (BMI), American Society of Anaesthesiologists (ASA) classification, smoking status, surgical indication, and history of previous spinal surgery. Surgical data included the number of levels operated on, use of fixation, surgical urgency, and the use of local bone grafts.

Surgical outcomes included the incidence of postoperative complications, the need for reoperation, and the length of hospital stay. PROMs were assessed preoperatively and at one year postoperatively. PROMs comprised the numeric rating scale (NRS) for arm pain and the European myelopathy score (EMS) [12].

### Study participants

The Swespine database was searched for all patients who underwent ACDF procedures with or without plates and bone grafts between 2006 and 2020. Adult patients (> 18 years old) with at least one year of follow-up clinical outcome data and PROMs were eligible for inclusion. Patients who underwent ACDF using crista bone, other graft materials or with unspecified graft types, as well as those with incomplete baseline or outcome data, were excluded from the analysis. Patients treated with a crista graft have been assessed in a previous publication [10].

### Outcomes

The primary outcome was the proportion of patients achieving a minimal clinically important difference (MCID) when comparing patients who received a local bone graft with those who did not. Among patients treated for radiculopathy, MCID was defined as an improvement of ≥ 3 points on the NRS for arm pain, based on previously published literature [9]. For patients with myelopathy, MCID was defined using the minimal detectable change (MDC) of the EMS, calculated according to established methodology [15]. Secondary outcomes included differences between the two groups in complication rates, reoperation rates, and length of hospital stay.

### Statistical analysis

Continuous variables are presented as means with standard deviations, and categorical variables as counts with percentages. Binary outcomes, including MCID achievement, complications, and reoperations, were assessed using multivariable generalized linear models (GLM) with a binomial family. Length of stay was evaluated using a multivariable GLM with a Gaussian family. Surgical extent (single and multi-level) and plate use (no plate and plate) were included as covariates in the MCID achievement models, while surgical indication (radiculopathy, myelopathy, and other), surgical extent, and plate use were included in the remaining models to enhance interpretability and reduce confounding. Covariates were selected based on their known influence on baseline characteristics and postoperative outcomes in ACDF [2, 3, 32, 33]. A *p*-value of < 0.05 was considered statistically significant. All statistical analyses and graph generation were conducted in R statistical software (version 4.2.3) [25].

### Ethical considerations

Informed consent was waived for inclusion in the Swespine registry due to the use of an opt-out model. Participation in follow-up questionnaires is voluntary. The study was approved by the Swedish Ethical Review Authority (Dnr: 2020–00193 and 2021–04773).

## Results

### Baseline patient characteristics

Of the 12,839 patients recorded in the Swespine registry, 905 were excluded due to missing procedural data. An additional 5,364 patients were excluded either because they did not undergo ACDF or because ACDF was performed using crista bone, alternative, or unspecified graft materials. Consequently, 6,571 patients were retained for analysis. The patients were categorized into two groups: 3,608 patients (55%) who received cages without local bone grafts and 2,963 patients (45%) who received cages packed with local bone grafts.

Baseline demographic and clinical characteristics were similar between patients treated with and without local bone graft (Table 1). There were no notable differences in age (50.8 ± 10.2 years vs. 51.0 ± 9.8 years), sex distribution (male: 48.2% vs. 49.9%) BMI (27.2 ± 4.0 vs. 27.1 ± 4.1), smoking status (13.2% vs. 13.6%), or admission type (elective: 97.4% vs. 97.3%). ASA classification and preoperative PROMs, including NRS arm pain (5.6 ± 2.7 vs. 5.7 ± 2.6) and EMS (15.2 ± 2.6 vs. 15.3 ± 2.5), were comparable between groups.

**Table 1:** Baseline demographic and clinical characteristics, stratified by use of local bone graft

Characteristic	Without local bone graft (*n* = 3,608)	With local bone graft (*n* = 2,963)
Age (years), mean (SD)	50.8 (10.2)	51.0 (9.8)
Male Sex, *n* (%)	1,740 (48.2%)	1,479 (49.9%)
Body Mass Index (BMI), mean (SD)	27.2 (4.0)	27.1 (4.1)
Smoker, *n* (%)	475 (13.2%)	402 (13.6%)
ASA Class, *n* (%)		
1	679 (48.4%)	498 (45.0%)
2	638 (45.5%)	543 (49.1%)
3	85 (6.1%)	66 (6.0%)
4	1 (0.1%)	0 (0%)
Previous spine surgery, *n* (%)	297 (8.2%)	273 (9.2%)
Admission setting, *n* (%)		
Elective	3,515 (97.4%)	2,883 (97.3%)
Non-elective	93 (2.6%)	80 (2.7%)
Fixation used, *n* (%)	3,562 (98.7%)	2,957 (99.8%)
Indication		
Radiculopathy	2,924 (81.4%)	2,499 (85.6%)
Myelopathy	635 (17.7%)	419 (14.3%)
Others	34 (0.9%)	3 (0.1%)
Level		
Single Level	2,439 (67.6%)	1,893 (63.9%)
Multi-Level	1,169 (32.4%)	1,070 (36.1%)
Plate Use		
Without Plate	1,846 (51.2%)	1,505 (50.8%)
With Plate	1,762 (48.8%)	1,458 (49.2%)
Preoperative NRS arm, mean (SD)	5.6 (2.7)	5.7 (2.6)
Preoperative EMS, mean (SD)	15.2 (2.6)	15.3 (2.5)

*ASA* American Society of Anaesthesiologists, *BMI* Body mass index, *MCID* Minimal clinically important difference, *NRS* Numeric rating scale, *EMS* European myelopathy score

Fixation was used in nearly all procedures, though slightly more frequent in the bone graft group (98.7% vs. 99.8%). Patients treated with local graft material had a slightly higher proportion of multilevel procedures (32.4% vs. 36.1%) and radiculopathy as the primary surgical indication (81.4% vs. 85.6%). Other characteristics, including prior spine surgery history (8.2% vs. 9.2%) and plate usage (48.8% vs. 49.2%), were comparable between the groups.

### Postoperative outcomes

**Fig. 1:**
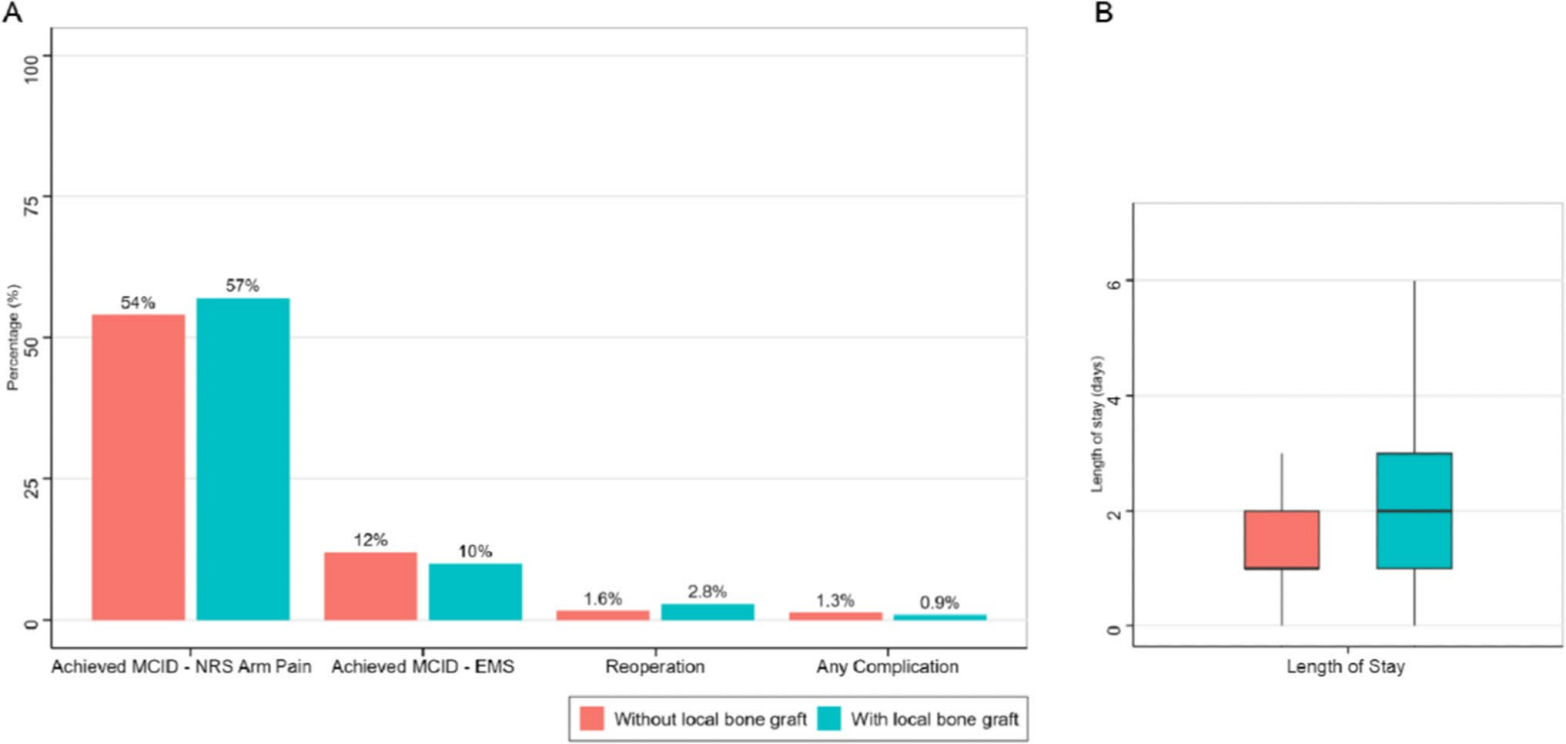
Comparison of patient-reported and surgical outcomes between ACDF procedures performed with and without local bone graft. **A** Proportion of patients achieving a minimal clinically important difference (MCID) in numeric rating scale (NRS) arm pain (radiculopathy subgroup) and European myelopathy score (EMS) (myelopathy subgroup), as well as rates of reoperation and any complication. **B** Distribution of postoperative length of stay shown as boxplots for both groups

Use of local bone graft was not associated with postoperative improvement in PROMs (Table 2). Among patients with radiculopathy, the odds of achieving MCID on the NRS arm pain scale did not differ between groups (OR 0.90, 95% CI 0.79–1.04, *p* = 0.15). In patients with myelopathy, local bone graft use was not associated with achieving MCID on the EMS (OR 1.15, 95% CI 0.67–2.03, *p* = 0.62) (Fig. 1).

**Table 2:** Association between bone graft use and patient-reported and surgical outcomes. Multivariable logistic regression models were used for binary outcomes and a multivariable linear regression model for length of stay. All models were adjusted for number of operated levels and plate use. Indication was additionally included as a covariate in the complication, reoperation, and length-of-stay models. The group without a local bone graft served as the reference category in all models

Characteristic	Odds Ratio (OR)	95% CI	*p*
MCID Achievement, NRS arm pain (Radiculopathy subgroup)	0.90	0.79—1.04	*0.15*
MCID Achievement, EMS (Myelopathy subgroup)	1.15	0.67—2.03	*0.62*
Complications	0.73	0.45—1.17	*0.20*
Reoperations	1.69	1.20—2.39	*0.003*
	**Coefficient (Beta)**	**95% CI**	***p***
Length of stay	0.31	0.21—0.40	< *0.001*

*CI* Confidence Interval, *OR* Odds Ratio, *MCID* Minimal clinically important difference, *NRS* Numeric rating scale, *EMS* European myelopathy score

Regarding surgical outcomes, complication rates were comparable between groups (OR 0.73, 95% CI 0.45–1.17, *p* = 0.20). In contrast, patients receiving local bone grafts had higher odds of reoperation compared with those without graft use (OR 1.69, 95% CI 1.20–2.39, *p* = 0.003). Length of hospital stay was also longer in the bone graft group, with a significant adjusted mean increase of 0.31 days (β = 0.31, 95% CI 0.21–0.40, *p* < 0.001).

## Discussion

Anterior cervical discectomy and fusion (ACDF) is one of the most commonly performed spinal procedures, yet there is considerable variability in the choice of graft materials and cage constructs. Using local bone as a graft material has been shown to be both effective and cost-efficient compared to other alternatives [14, 17]. However, clinical outcomes with respect to the use of local bone grafts in cages during ACDFs remain understudied, and comparisons with patients receiving cages without local bone grafts are limited. A survey of 599 spine surgeons found that only 54.5% expressed satisfaction with the available comparative effectiveness data on bone graft materials for ACDF, highlighting the need for further research on this topic [35].

In our nationwide registry-based analysis of 6,571 ACDF patients in Sweden, we found that both local bone grafts and unfilled cages were commonly used. Our findings indicate that both approaches yield comparable outcomes, with no meaningful differences in surgical or patient-reported outcome measures (PROM). While we did not assess radiological fusion rates, as this information is not present in Swespine and many patients do not receive long-term radiological follow-up unless clinically indicated, we intentionally focus on PROMs as opposed to radiological outcomes or surgeon-reported outcome scales. In recent decades, the shift of importance in research has gone from focusing on radiological outcomes to PROMs – and, in the end, even if there would be a difference in fusion rates, but no difference in PROMs, the relevance of such radiographic findings may be questionable. By additionally evaluating the minimally clinically important difference (MCID), our analysis moves beyond statistical comparisons of PROM means and instead focuses on whether patients experienced a clinically relevant improvement. In this respect, we can safely say that, based on highly covered national data, the outcome profile of both implant techniques for ACDF is equivalent.

A secondary finding of this study was that the use of local bone grafts was independently associated with longer hospital stays. Although the precise cause of this difference is unclear and cannot be mechanistically explained or causality postulated based on our registry data, it may potentially be related to postoperative pain. The Swespine registry collects pain data as a PROM at one year postoperatively, which limits our ability to directly correlate pain levels with length of stay. Although a difference of 0.31 days may not have a meaningful impact on an individual level, it could have significant implications for healthcare systems due to associated costs. Still, it is questionable whether the difference is only statistically relevant, as it certainly does not represent a major clinically relevant difference. Another limitation to consider is that, in the Swespine registry, length of stay is calculated based on admission and discharge dates without accounting for the exact times of hospitalization. This approach may lead to minor inaccuracies and further limits the robustness of the findings. In contrast to our findings, Kanna et al. compared functional and radiological outcomes between patients receiving allografts and local bone grafts in a cohort of 27 patients, finding no significant differences in hospital stay, functional, or radiological outcomes [14]. In our study, although local bone graft use was associated with higher odds for reoperation in the adjusted analysis (OR 1.69, 95% CI 1.20–2.39), the absolute difference between groups was small (1.6% vs. 2.8%). This finding differs from a prior literature review and network meta-analysis, which reported no significant differences in reoperation rates between standalone cages and cages supplemented with graft material [19]. In their review of studies, the authors noted that common indications for revision of ACDF included pseudoarthrosis, infection, progressive deformity, adjacent segment disease, or their combinations [19].

By analyzing MCID in addition to conventional surgical outcomes and adjusting for key clinical confounders (surgical indication, extent, and plate use), our study provides a robust, patient-focused comparison and demonstrates that both techniques perform equivalently with respect to long-term outcomes. Several additional analyses would be of interest, namely if the type of local grafting method could perhaps still have an impact on outcomes. For example, different types of local bone grafting (bone dust vs. osteophyte bone, and vertebral body reams) could constitute unmeasured surgical confounders [6, 21]. Further studies may delve into such more detailed analyses to confirm the results of smaller studies on a larger, post-market analysis level based on national registry data with high coverage.

Complication rates and MCID achievement for NRS arm pain and EMS showed no significant association with local bone graft use in either radiculopathy or myelopathy patients. While previous studies have reported good outcomes with local bone grafts, the literature on outcomes after the use of empty cages remains inconsistent. For example, the PIERCE-PEEK study, a prospective multicenter study on ACDF with empty PEEK cages, found that the overall rate of radiographic fusion with empty PEEK cages was slow and insufficient, which may lead to less improvement in pain and disability [30]. Conversely, a study by Feng et al. in a randomized setting showed that patients receiving either empty cages or cages filled with β-tricalcium phosphate had similar fusion rates and clinical outcomes [11]. Our findings align more closely with the results of Feng et al. as any potential differences in clinical outcomes were small and did not translate into meaningful differences in MCID achievement. Ultimately, while radiographic and conventional surgical outcomes are important, the most crucial factor is the patient's perspective. In this regard, our analysis indicates that patient-centered outcomes were comparable, regardless of whether an empty cage or a cage supplemented with local bone graft was used.

## Limitations

This study has several limitations that must be considered. First, bias from being a retrospective observational design study cannot be excluded. Second, reporting biases associated with the use of PROMs may also affect the results. Third, detailed information regarding the types of cages used or the type of local bone graft (e.g., local bone dust or bone from osteophytes) is lacking. However, due to the nature of the registry, this was not possible to obtain in a reliable way. In fact, in general, a weakness of registry studies is the fact that granular, procedure-specific data are often not available. This also, for example, precludes us from delving into potential mechanistic links and confounders for secondary outcomes (length of stay, reoperation rate), and thus rather only allows us to describe more superficial conclusions on safety and effectiveness – but, with epidemiologically strong data with a high degree of national coverage. Due to the nature of the registry, obtaining this data in a reliable manner was not possible. This also counts for data such as bone quality or radiological fusion rates, which could have relevance for analysis but are unavailable. Future studies should aim to collect this granular data and consider performing a cost-effectiveness analysis of the two groups.

## Conclusion

In this study of 6,571 patients from the Swedish Spine Registry, we found that the surgical outcomes of ACDF performed with or without local bone autograft were largely comparable. ACDF performed with or without local bone graft is equally effective, with comparable rates of patient-reported outcome MCID achievement and similar adverse event profiles, although use of local bone graft was associated with higher reoperations and longer hospital stays. Local bone grafting thus does not seem to provide a clinically relevant advantage to the patient, with potentially higher associated harms.

## Supplementary Information

Below is the link to the electronic supplementary material.

ESM 1 Supplementary Material 1 (DOCX 28.9 KB)

ESM 2 Supplementary Material 2 (DOCX 26.4 KB)

## Data Availability

The data in support of our findings can be obtained upon reasonable request from the corresponding author.
