# Supplementary material for: A nationwide registry study of surgical and patient-reported outcomes following anterior cervical discectomy and fusion: Part 2 – cage with versus without local bone graft
Source: Acta Neurochir (Wien). 2025 Dec 11;167(1):319. doi: 10.1007/s00701-025-06751-w (PMC12700931; doi:10.1007/s00701-025-06751-w)
Supplement: Supplementary file 1 — Supplementary Material 1 (DOCX 28.9 KB) [file 701_2025_6751_MOESM1_ESM.docx]

|  | **Without local bone graft**  (n = 3,608) | | | | | | | **With local bone graft**  (n = 2,963) | | | | | | |
| --- | --- | --- | --- | --- | --- | --- | --- | --- | --- | --- | --- | --- | --- | --- |
| **Characteristic** | **Overall** | **Radiculopathy**  (n = 2924) | **Myelopathy**  (n = 635) | **Single Level**  (n = 2439) | **Multi-Level**  (n = 1169) | **Without plate**  (n = 1846) | **With plate**  (n = 1762) | **Overall** | **Radiculopathy**  (n = 2499) | **Myelopathy**  (n = 419) | **Single Level**  (n = 1893) | **Multi-Level**  (n = 1070) | **Without plate**  (n = 1505) | **With plate**  (n = 1458) |
| **Age (years), mean (SD)** | 50.8 (10.2) | 49.8 (9.6) | 55.7 (11.4) | 50.0 (10.5) | 52.5 (9.2) | 50.7 (10.3) | 51.0 (10.0) | 51.0 (9.8) | 49.9 (9.1) | 56.9 (11.8) | 50.0 (10.2) | 52.7 (9.0) | 50.1 (9.8) | 51.9 (9.8) |
| **Male Sex, n (%)** | 1,740 (48.2%) | 1,385 (47.4%) | 337 (53.1%) | 1,173 (48.1%) | 567 (48.5%) | 889 (48.2%) | 851 (48.3%) | 1,479 (49.9%) | 1,242 (49.7%) | 217 (51.8%) | 929 (49.1%) | 550 (51.4%) | 749 (49.8%) | 730 (50.1%) |
| **Body Mass Index (BMI), mean (SD)** | 27.2 (4.0) | 27.2 (4.0) | 27.2 (4.0) | 27.2 (4.0) | 27.2 (3.9) | 27.3 (4.0) | 27.1 (3.9) | 27.1 (4.1) | 27.1 (4.1) | 27.3 (4.3) | 27.1 (4.1) | 27.2 (4.1) | 27.0 (4.0) | 27.2 (4.2) |
| **Smoker, n (%)** | 475 (13.2%) | 398 (13.6%) | 74 (11.7%) | 316 (13.0%) | 159 (13.6%) | 268 (14.5%) | 207 (11.7%) | 402 (13.6%) | 333 (13.3%) | 57 (13.6%) | 266 (14.1%) | 136 (12.7%) | 213 (14.2%) | 189 (13.0%) |
| **ASA Class, n (%)** |  |  |  |  |  |  |  |  |  |  |  |  |  |  |
| 1 | 679 (48.4%) | 579 (50.4%) | 100 (39.4%) | 497 (49.8%) | 182 (44.8%) | 364 (50.7%) | 315 (46.0%) | 498 (45.0%) | 435 (48.2%) | 63 (30.7%) | 326 (49.4%) | 172 (38.5%) | 308 (53.9%) | 190 (35.4%) |
| 2 | 638 (45.5%) | 513 (44.6%) | 125 (49.2%) | 436 (43.7%) | 202 (49.8%) | 317 (44.2%) | 321 (46.9%) | 543 (49.1%) | 427 (47.3%) | 116 (56.6%) | 295 (44.7%) | 248 (55.5%) | 235 (41.2%) | 308 (57.5%) |
| 3 | 85 (6.1%) | 56 (4.9%) | 29 (11.4%) | 64 (6.4%) | 21 (5.2%) | 37 (5.2%) | 48 (7.0%) | 66 (6.0%) | 40 (4.4%) | 26 (12.7%) | 39 (5.9%) | 27 (6.0%) | 28 (4.9%) | 38 (7.1%) |
| 4 | 1 (0.1%) | 1 (0.1%) | 0 (0%) | 0 (0%) | 1 (0.2%) | 0 (0%) | 1 (0.1%) | 0 (0%) | 0 (0%) | 0 (0%) | 0 (0%) | 0 (0%) | 0 (0%) | 0 (0%) |
| **Admission setting, n (%)** |  |  |  |  |  |  |  |  |  |  |  |  |  |  |
| Elective | 3,515 (97.4%) | 2,863 (97.9%) | 603 (95.0%) | 2,369 (97.1%) | 1,146 (98.0%) | 1,813 (98.2%) | 1,702 (96.6%) | 2,883 (97.3%) | 2,448 (98.0%) | 392 (93.6%) | 1,829 (96.6%) | 1,054 (98.5%) | 1,465 (97.3%) | 1,418 (97.3%) |
| Non-elective | 93 (2.6%) | 61 (2.1%) | 32 (5.0%) | 70 (2.9%) | 23 (2.0%) | 33 (1.8%) | 60 (3.4%) | 80 (2.7%) | 51 (2.0%) | 27 (6.4%) | 64 (3.4%) | 16 (1.5%) | 40 (2.7%) | 40 (2.7%) |
| **Fixation used, n (%)** | 3,562 (98.7%) | 2,891 (98.9%) | 623 (98.1%) | 2,404 (98.6) | 1,158 (99.1%) | 1,806 (97.8%) | 1,756 (99.7%) | 2,957 (99.8%) | 2,493 (99.8%) | 419 (100.0%) | 1,888 (99.7%) | 1,069 (99.9%) | 1,500 (99.7%) | 1,457 (99.9%) |
| **Indication** |  |  |  |  |  |  |  |  |  |  |  |  |  |  |
| Radiculopathy | 2,924 (81.4%) | 2,924 (100%) | 0 (0%) | 2,052 (84.6%) | 872 (74.7%) | 1,563 (85.0%) | 1,361 (77.5%) | 2,499 (85.6%) | 2,499 (100%) | 0 (0%) | 1,625 (87.3%) | 874 (82.5%) | 1,315 (88.3%) | 1,184 (82.7%) |
| Myelopathy | 635 (17.7%) | 0 (0%) | 635 (100%) | 356 (14.7%) | 279 (23.9%) | 262 (14.3%) | 373 (21.3%) | 419 (14.3%) | 0 (0%) | 419 (100%) | 234 (12.6%) | 185 (17.5%) | 174 (11.7%) | 245 (17.1%) |
| Others | 34 (0.9%) | 0 (0%) | 0 (0%) | 18 (0.7%) | 16 (1.4%) | 13 (0.7%) | 21 (1.2%) | 3 (0.1%) | 0 (0%) | 0 (0%) | 3 (0.2%) | 0 (0%) | 1 (0.1%) | 2 (0.1%) |
| **Level** |  |  |  |  |  |  |  |  |  |  |  |  |  |  |
| Single Level | 2,439 (67.6%) | 2,052 (70.2%) | 356 (56.1%) | 2,439 (100%) | 0 (0%) | 1,485 (80.4%) | 954 (54.1%) | 1,893 (63.9%) | 1,625 (65.0%) | 234 (55.8%) | 1,893 (100%) | 0 (0%) | 1,195 (79.4%) | 698 (47.9%) |
| Multi-Level | 1,169 (32.4%) | 872 (29.8%) | 279 (43.9%) | 0 (0%) | 1,169 (100%) | 361 (19.6%) | 808 (45.9%) | 1,070 (36.1%) | 874 (35.0%) | 185 (44.2%) | 0 (0%) | 1,070 (100%) | 310 (20.6%) | 760 (52.1%) |
| **Plate Use** |  |  |  |  |  |  |  |  |  |  |  |  |  |  |
| Without Plate | 1,846 (51.2%) | 1,563 (53.5%) | 262 (41.3%) | 1,485 (60.9%) | 361 (30.9%) | 1,846 (100%) | 0 (0%) | 1,505 (50.8%) | 1,315 (52.6%) | 174 (41.5%) | 1,195 (63.1%) | 310 (29.0%) | 1,505 (100%) | 0 (0%) |
| With Plate | 1,762 (48.8%) | 1,361 (46.5%) | 373 (58.7%) | 954 (39.1%) | 808 (69.1%) | 0 (0%) | 1,762 (100%) | 1,458 (49.2%) | 1,184 (47.4%) | 245 (58.5%) | 698 (36.9%) | 760 (71.0%) | 0 (0%) | 1,458 (100%) |
| **Preoperative NRS arm, mean (SD)** | 5.57 (2.7) | 5.69 (2.58) | 5.01 (2.97) | 5.59 (2.68) | 5.53 (2.65) | 5.66 (2.58) | 5.47 (2.76) | 5.71 (2.62) | 5.84 (2.55) | 4.89 (2.92) | 5.68 (2.62) | 5.76 (2.61) | 5.61 (2.64) | 5.82 (2.60) |
| **Preoperative EMS, mean (SD)** | 15.19 (2.61) | 15.42 (2.41) | 14.13 (3.19) | 15.24 (2.58) | 15.10 (2.67) | 15.21 (2.55) | 15.17 (2.67) | 15.33 (2.52) | 15.56 (2.36) | 13.86 (3.05) | 15.35 (2.53) | 15.28 (2.52) | 15.44 (2.47) | 15.21 (2.58) |

**Suppl. Table 1.** Baseline demographic and clinical characteristics, stratified by use of local bone graft. Data are presented overall and stratified by indication (radiculopathy vs myelopathy), surgical extent (single level vs multilevel), and plate use (with vs without plate) for both groups.

*Abbreviations: ASA =* *American Society of Anaesthesiologists, BMI = Body mass index, MCID = Minimal clinically important difference, NRS = Numeric rating scale, EMS = European myelopathy score*
