# Supplementary material for: A nationwide registry study of surgical and patient-reported outcomes following anterior cervical discectomy and fusion: Part 2 – cage with versus without local bone graft
Source: Acta Neurochir (Wien). 2025 Dec 11;167(1):319. doi: 10.1007/s00701-025-06751-w (PMC12700931; doi:10.1007/s00701-025-06751-w)
Supplement: Supplementary file 2 — Supplementary Material 2 (DOCX 26.4 KB) [file 701_2025_6751_MOESM2_ESM.docx]

| **Characteristic** | **Odds Ratio (OR)** | **95% CI** | ***p*** |
| --- | --- | --- | --- |
| **MCID Achievement, NRS arm pain (Radiculopathy subgroup)** | 0.90 | 0.79 - 1.04 | *0.15* |
| Intercept | 0.83 | 0.73 - 0.93 | *0.002* |
| Level (ref. single level) | 1.05 | 0.90 - 1.22 | *0.55* |
| Plate Use (ref. no plate) | 1.02 | 0.88 - 1.18 | *0.79* |
| **MCID Achievement, EMS**  **(Myelopathy subgroup)** | 1.15 | 0.67 - 2.03 | *0.62* |
| Intercept | 6.40 | 4.04 - 10.6 | *<0.001* |
| Level (ref. single level) | 1.11 | 0.64 - 1.94 | *0.72* |
| Plate Use (ref. no plate) | 1.22 | 0.69 - 2.12 | *0.49* |
| **Complications** | 0.73 | 0.45 - 1.17 | *0.20* |
| Intercept | 0.01 | 0.01 - 0.02 | *<0.001* |
| Indication (ref. radiculopathy) | 1.21 | 0.66 - 2.10 | *0.51* |
| Level (ref. single level) | 1.41 | 0.86 - 2.29 | *0.17* |
| Plate Use (ref. no plate) | 1.17 | 0.72 - 1.90 | *0.54* |
| **Reoperations** | 1.69 | 1.20 - 2.39 | *0.003* |
| Intercept | 0.02 | 0.01 - 0.02 | *<0.001* |
| Indication (ref. radiculopathy) | 0.98 | 0.60 - 1.53 | *>0.9* |
| Level (ref. single level) | 1.21 | 0.84 - 1.74 | *0.30* |
| Plate Use (ref. no plate) | 1.02 | 0.71 - 1.46 | *>0.9* |
|  | **Coefficient (Beta)** | **95% CI** | ***p*** |
| **Length of stay** | 0.31 | 0.21 - 0.40 | *<0.001* |
| Intercept | 1.8 | 1.7 - 1.9 | *<0.001* |
| Indication (ref. radiculopathy) | 0.31 | 0.18 - 0.45 | *<0.001* |
| Level (ref. single level) | 0.29 | 0.18 - 0.39 | *<0.001* |
| Plate Use (ref. no plate) | -0.13 | -0.23 - -0.03 | *0.013* |
| *Abbreviations: CI = Confidence Interval, OR = Odds Ratio, MCID = Minimal clinically important difference, NRS = Numeric rating scale, EMS = European myelopathy score* | | | |

**Suppl. Table 2.** Association between bone graft use and patient-reported and surgical outcomes. Multivariable logistic regression models were used for binary outcomes and a multivariable linear regression model for length of stay. All models were adjusted for number of operated levels and plate use. Indication was additionally included as a covariate in the complication, reoperation, and length-of-stay models. For each model, the first line reflects the adjusted effect of bone graft use compared with no graft. Additional rows list the effects of covariates included in the model. The group without a local bone graft served as the reference category in all models.
